# Pancreatic stellate cell secreted IL-6 stimulates STAT3 dependent invasiveness of pancreatic intraepithelial neoplasia and cancer cells

**DOI:** 10.18632/oncotarget.11786

**Published:** 2016-09-01

**Authors:** Nagaraj S. Nagathihalli, Jason A. Castellanos, Michael N. VanSaun, Xizi Dai, Mahogany Ambrose, Qiaozhi Guo, Yanhua Xiong, Nipun B. Merchant

**Affiliations:** ^1^ Division of Surgical Oncology, Department of Surgery, University of Miami Miller School of Medicine, Sylvester Comprehensive Cancer Center, Miami, Florida, USA; ^2^ Department of Surgery, Vanderbilt University School of Medicine, Nashville, Tennessee, USA; ^3^ Meharry Medical College, Nashville, Tennessee, USA; ^4^ Yale School of Medicine, New Haven, Connecticut, USA

**Keywords:** pancreatic stellate cells, IL-6, STAT3, PanIN, pancreatic cancer

## Abstract

Pancreatic ductal adenocarcinoma (PDAC) is a dynamic tumor supported by several stromal elements such as pancreatic stellate cells (PSC). Significant crosstalk exists between PSCs and tumor cells to stimulate oncogenic signaling and malignant progression of PDAC. However, how PSCs activate intercellular signaling in PDAC cells remains to be elucidated. We have previously shown that activated signal transducer and activator of transcription 3 (STAT3) signaling is a key component in the progression of pancreatic neoplasia. We hypothesize that PSC secreted IL-6 activates STAT3 signaling to promote PanIN progression to PDAC. Human PDAC and mouse PanIN cells were treated with PSC-conditioned media (PSC-CM), and phospho- and total-STAT3 levels by immunoblot analysis were determined. IL-6 was quantified in PSC-CM and cell invasion and colony formation assays were performed in the presence or absence of a neutralizing IL-6 antibody and the JAK/STAT3 inhibitor AZD1480. Serum from *Ptf1a^Cre/+^;LSL-Kras^G12D/+^;Tgfbr2^flox/flox'^* (PKT) and *LSL-Kras^G12D/+^; Trp53^R172H/+^; Pdx1^Cre/+^* (KPC) mice demonstrated increased levels of IL-6 compared to serum from non-PDAC bearing KC and PK mice. PSC secreted IL-6 activated STAT3 signaling in noninvasive, precursor PanIN cells as well as PDAC cells, resulting in enhanced cell invasion and colony formation in both cell types. There was a significant positive linear correlation between IL-6 concentration and the ratio of phosphorylated STAT3/total STAT3. IL-6 neutralization or STAT3 inhibition attenuated PSC-CM induced activation of STAT3 signaling and tumorigenicity. These data provide evidence that PSCs are directly involved in promoting the progression of PanINs towards invasive carcinoma. This study demonstrates a novel role of PSC secreted IL-6 in transitioning noninvasive pancreatic precursor cells into invasive PDAC through the activation of STAT3 signaling.

## INTRODUCTION

The extremely poor outcomes associated with pancreatic ductal adenocarcinoma (PDAC) are related to its high frequency of genetic mutations, existence of interdependent signaling pathways, and the presence of a dense stromal compartment encapsulating the tumor [1, 2] [3]. Elevated tumor interstitial fluid pressure and decreased vascularity is partly responsible for the poor penetration and delivery of therapeutic agents in PDAC [4]. Emerging evidence indicates that the extensive stroma of PDAC not only impedes drug delivery, but also plays a role in promoting tumor growth, facilitating invasion and metastasis, and mediating therapeutic resistance of PDAC [5, 6]. Recent evidence indicates that crosstalk between the tumor and stromal elements of the tumor microenvironment (TME) mediates cancer progression and metastasis [7]. The pancreatic tumor-associated stroma consists of several cell types, including immune cells, endothelial cells and stellate cells. These stromal cell types display a dynamic interaction with each other, as well as with PDAC cells [5, 6]. Precursor pancreatic intraepithelial neoplasia (PanIN) lesions are known to progress and develop into invasive PDAC. A reactive stroma accompanies this transition from PanIN, resulting in a fibrotic desmoplasia with a hallmark of immune cell infiltration [8]. However, the mechanism through which tumor-associated stromal cells promote the growth and progression of PDAC from PanINs is not well understood.

The myofibroblast-like pancreatic stellate cells (PSCs) reside in a quiescent state in the normal pancreas, but transition to an activated state under pathological conditions such as inflammation or cancer [5, 6, 9, 10]. Numerous studies have shown that PDAC is often associated with the infiltration of immune cells that creates an inflamed microenvironment enriched with growth factors, cytokines, endotoxins, a hypoxic TME and an increased oxidant stress level, all of which contribute to the activation of PSCs [11, 12]. Yet, how PSCs activate intercellular signaling in PDAC cells remains to be elucidated.

Cytokines represent therapeutically targetable mediators of PDAC progression. Specifically, IL-6 is a multifunctional cytokine that has been shown to be elevated in the serum of patients with PDAC [13] and confirmed to promote PDAC development in a mouse model [14]. STAT3, a downstream signaling mediator of IL-6, is activated in stromal and myeloid cells and is a critical element in the formation of a pre-metastatic niche, leading to increased metastasis [15–17]. We have previously shown that the activated STAT3 correlates with poor prognosis in patients with PDAC [18–20]. Previous studies have shown that IL-6 from macrophages and myeloid cells in the TME activate STAT3 signaling in PDAC [14] [21]. We have demonstrated a mechanistic rationale for activated STAT3 as a biomarker of therapeutic resistance in PDAC [1, 2]. The level of STAT3 expression in PDAC cell lines correlates with resistance to treatment, and the level of phosphorylated STAT3 expression in human pancreatic tissues correlates positively with PDAC progression and negatively with overall survival [20]. These data confirm the functional role of activated STAT3 signaling in the development of PDAC.

The purpose of this study was to determine the influence of cytokine-induced signaling between PSCs and neoplastic cells at the earliest stages of pancreatic cancer development. This study demonstrates for the first time that PSCs actively secrete IL-6, which in turn activates STAT3 signaling in PanIN cells, and promotes the invasive and tumorigenic capacity of these pancreatic precursor lesions as well as already established PDAC cells.

## RESULTS

### Phosphorylation of STAT3 is increased with the progression of PDAC

**Figure 1:**
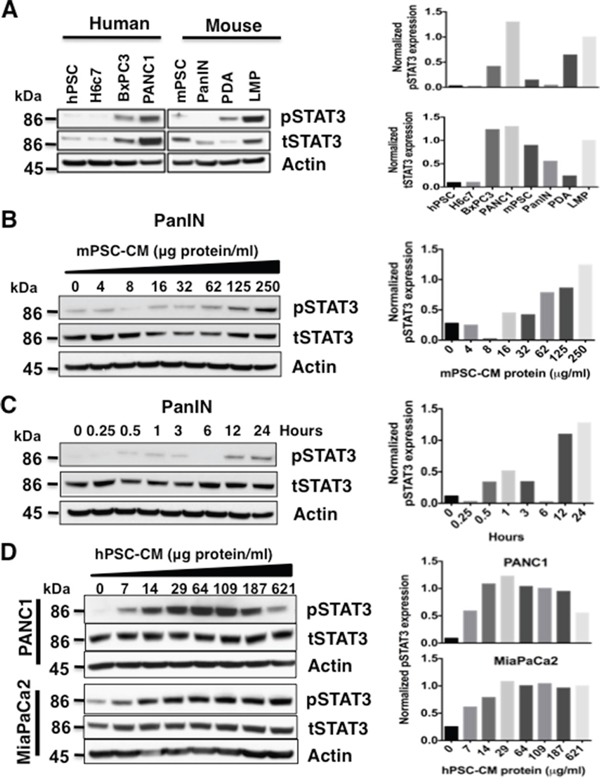
Pancreatic stellate cell (PSC) conditioned media (CM) activates STAT3 **A.** Baseline STAT3 level was obtained by immunoblotting total cell lysates from human (left panel) PSC (hPSCs), pancreatic ductal cells (H6c7), and PDAC cells lines (BxPC3, PANC1), as well as mouse (right panel) PSC (mPSCs), PanIN, PDA and liver metastasis (LMP) cell lines. Only the tumor cell lines expressed high levels of pSTAT3 at baseline. Densitometry analyses for the immunoblots from pSTAT3 (top panel) and tSTAT3 (bottom panel) after normalization to actin protein is shown to the right. **B** and **C.** Serial exposure to concentrated mPSC-CM results in activation of STAT3 in mouse PanIN cells in a dose- (B) and time- (C) dependent manner. Densitometry analyses of (B) and (C) pSTAT3 immunoblots normalized to tSTAT3 are shown in the graphs to the right. **D.** PANC1 and MiaPaCa2 cells also demonstrate a dose- dependent increase in STAT3 activation when exposed to hPSC-CM (left panel). Densitometry analyses of pSTAT3 immunoblots normalized to tSTAT3 for PANC1 (top panel) and MiaPaCa2 (bottom panel) cells are displayed in graphs to the right.

The baseline expression of total and phospho-STAT3 (pSTAT3) (Figure 1, left panel) was examined in a human pancreatic stellate cell line (hPSC), immortalized pancreatic ductal epithelial cell line HPDE6-E6E7 (H6c7), and in PDAC cell lines (BxPC3 and PANC1) (Figure 1A). Mouse pancreatic stellate cells (mPSC) and mouse PanIN cells from KC (*LSL-Kras^G12D/+^; Pdx1^Cre/+^*) mice, primary PDAC (PDA) and liver metastasis (LMP) cell lines derived from the KPC (*LSL-Kras^G12D/+^; Trp53^R172H/+^; Pdx1^Cre/+^*) mice [22] were also examined for baseline total and pSTAT3 expression (Figure 1A, right panel). As shown in Figure 1A, pSTAT3 levels were low to undetectable in normal and benign pancreatic epithelial cells and PanIN cells, but increased with the progression of pancreatic neoplasia from PanIN cells to primary PDAC to liver metastasis in both human and mouse cell lines.

### Pancreatic stellate cell - conditioned medium (PSC-CM) protein activates STAT3 signaling in PanIN and PDAC cells

PSCs are known to produce numerous cytokines, which in turn can regulate oncogenic signaling pathways. To study the role of PSCs in activating STAT3 signaling, mouse PanIN cells were exposed to mouse PSC-CM (mPSC-CM) concentrates in a dose- (0-250 μg protein/ml) and time- (0-24 hours, 50 μg protein/ml) dependent manner (Figure 1B and 1C). Phosphorylation of STAT3 in these cell lysates was determined by western blot. Serial exposure to concentrated mPSC-CM in PanIN cells and human PSC-CM (hPSC-CM, 0-621 μg/ml) in human PDAC PANC1 and MiaPaCa2 cells increased pSTAT3 levels in both a concentration (Figure 1B and 1D) and time-dependent (Figure 1C and Supplementary Figure S1) manner. Downregulation of pSTAT3 expression in PANC1 cells treated with 621 μg protein/ml hPSC-CM protein occurred in the last lane, suggesting an effective cell type specific concentration window.

### PSC secreted IL-6 and serum IL-6 is increased in Ptf1a^Cre/+^;LSL-Kras^G12D/+^;Tgfbr2^flox/flox^ (PKT) and LSL-Kras^G12D/+^; Trp53^R172H/+^; Pdx1^Cre/+^ (KPC) mice

**Figure 2:**
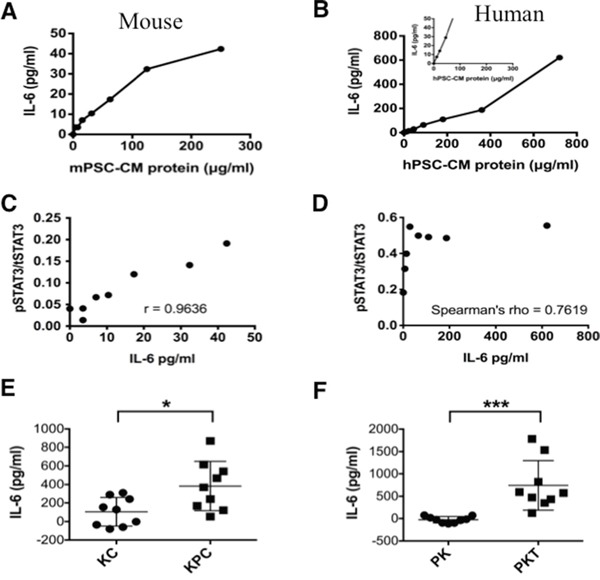
IL-6 concentration in mouse and human PSC-CM Levels of IL-6 were measured by ELISA relative to total protein levels in serially diluted CM secreted from mPSCs **A.** and hPSCs **B.** Inset for hPSCs (B) shows the same concentration range as provided for the mPSCs (A). The Relationship between recombinant IL-6 concentration and STAT3 activation; **C.** The ratio of pSTAT3 to tSTAT3 levels in mouse PanIN cells was determined by quantifying pSTAT3 and tSTAT3 luminescence on the immunoblots from Figure 1B (ImageJ) and performing a Pearson's correlation (p < 0.001). **D.** The relationship between IL-6 concentration and ratio of pSTAT3 to tSTAT3 levels in human MiaPaCa2 cells was determined by quantifying pSTAT3 and tSTAT3 luminescence on the immunoblots from Figure 1C (ImageJ) and performing a Spearman's correlation (p < 0.001). **E.** and **F.** *In vivo* analysis of IL-6 from the serum collected from *LSL-Kras^G12D/+^; Pdx1^Cre/+^* (KC) and *LSL-Kras^G12D/+^; Trp53^R172H/+^; Pdx1^Cre/+^* (KPC) mice (E) *or Ptf1a^Cre/+^*; *LSL-Kras^G12D/+^* (PK) and *Ptf1a^Cre/+^*; *LSL-Kras^G12D/+;^ Tgfbr2^flox/flox^* (PKT) mice (F). Serum from 3 mice was analyzed in triplicates (n=9). * – p<0.05; *** – P<0.001.

PSCs secrete factors that can support the growth and survival of tumor cells [23]. Cytokine array analysis of supernatants from PSCs showed significantly higher concentrations (1000-fold) of IL-6 compared with other cytokines (data not shown). IL-6 activates STAT3 signaling [24, 25]. We therefore studied the effects of PSC secreted IL-6 on STAT3 signaling in PanIN and PDAC cells. IL-6 levels were measured by ELISA in serially diluted samples of conditioned media (CM) collected from human and mouse PSCs (Figure 2). The concentration of PSC secreted IL-6 increased in concordance with the concentration of total protein content in the conditioned media. Overall, mPSC-CM (Figure 2A) demonstrated lower concentration ranges of IL-6 than hPSC-CM (Figure 2B) relative to total protein level. Exposure to higher concentrations of PSC-CM protein and therefore higher IL-6 concentrations, resulted in increased expression of pSTAT3 in PDAC cells (Figure 1D), except in PANC1 cells when pSTAT3 expression peaked at a relative IL-6 concentration of 64 μg/ml compared with 621 μg/ml in MiaPaCa2 cells.

Exposure of mouse PanIN cells to IL-6 resulted in a significant concentration-dependent positive linear association between the pSTAT3/tSTAT3 ratio and IL-6 concentration (Pearson's Correlation; r = 0.9636, p < 0.001, Figure 2C). MiaPaCa2 cells, which have a high baseline expression of pSTAT3 [20], also exhibited a significant, but nonlinear, dose response relationship between IL-6 exposure and pSTAT3/tSTAT3 ratio (Spearman's rho = 0.7619, p = 0.028, Figure 2D).

To further determine the systemic effects of IL-6 in the progression of pancreatic neoplasia, we compared the level of serum IL-6 in KC and PK mice (without PDAC) with those of KPC and PKT mice (with PDAC) respectively. Serum IL-6 levels were significantly higher in KPC (Figure 2E) and PKT (Figure 2F) mice when compared with their respective KC and PK control mice. In Figure 1A (right panel) we show that PDA and LMP lines derived from KPC mice have increased pSTAT3 expression compared with PanIN cells derived from KC mice, further corroborating the roles of IL-6 and activated STAT3 signaling in the progression of PDAC from PanINs.

### IL-6 secreted from PSCs activates STAT3 signaling in PDAC cells

**Figure 3:**
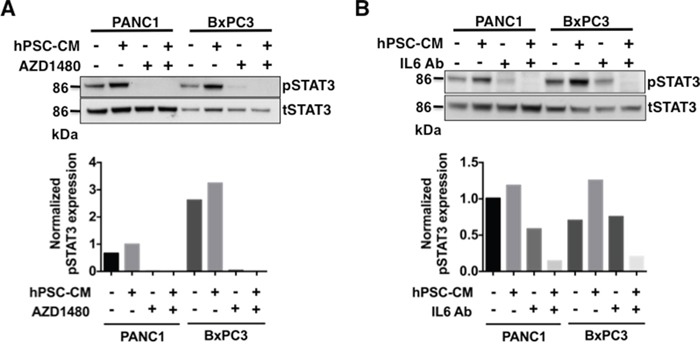
Pharmacological inhibition of JAK/STAT3 signaling or blocking IL-6 inhibits phosphorylation of STAT3 in hPSC-CM protein PDAC treated cells PANC1 and BxPC3 cells were treated with hPSC-CM with or without JAK/STAT3 inhibitor AZD1480 (100 nmol/L) **A.** or IL-6 neutralizing antibody **B.** At the end of the study, cell lysates were analyzed for total STAT3 and phospho-STAT3 levels by immunoblot analysis. Densitometry analyses of pSTAT3 normalized to tSTAT3 was shown in the bottom panels of A and B. AZD1480 or IL-6 Ab treatment inhibited hPSC-CM induced activation of STAT3.

To gain further insight into the ability of PSC secreted IL-6 to act as a critical mediator driving STAT3 activation in PDAC, PANC1 and BxPC3 cells were exposed to hPSC-CM with and without an IL-6 neutralizing antibody or the Jak/STAT3 inhibitor AZD1480. Pre-treatment of human PDAC cells with AZD1480 inhibited hPSC-CM (100μg protein/ml) mediated phosphorylation of STAT3 (Figure 3A). Treatment of hPSC-CM with an IL-6 neutralizing antibody effectively reduced the IL-6 concentration in the PSC-CM to IL-6 concentrations seen in serum-free control medium (Supplementary Figure S2). Exposure of IL-6 antibody-depleted hPSC-CM to PDAC cells also substantially reduced hPSC-CM mediated phosphorylation of STAT3 (Figure 3B). These results indicate PSC secreted IL-6 activates STAT3 signaling in PDAC cells.

### Neutralization of IL-6 abrogates PSC-CM induced cell invasion and anchorage independent growth

**Figure 4:**
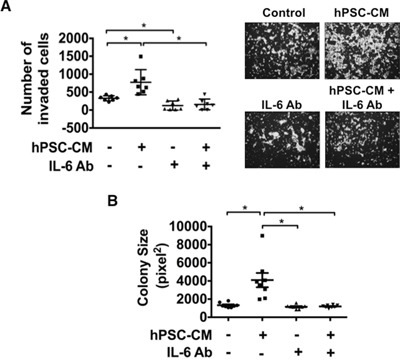
Blocking IL-6 attenuates hPSC-CM induced cell invasion and colony size PANC1 cells were treated with hPSC-CM (100μg total protein/ml) with or without IL-6 neutralizing antibody (IL-6 Ab). Cell invasion **A.** and colony size **B.** were analyzed as detailed in Materials and Methods. Cell invasion images demonstrate representative pictures from each treatment group (A, right panel). Cell invasion results represent the average number of cells in seven fields in triplicate inserts. Colony size results are represented by eight photographs analyzed from triplicate wells. * – P < 0.05.

STAT3 activation enhances the invasive ability of tumor cells [14, 26]. To determine if IL-6-mediated activation of STAT3 was able to enhance invasive ability of PDAC cells, PANC1 and BxPC3 cells were seeded in the upper chamber of a matrigel coated transwell filter with the lower chambers filled with hPSC-CM (100μg protein/ml) or control medium in the presence or absence of IL-6 neutralizing antibody (1:400 dilution). Exposure to hPSC-CM significantly enhanced invasiveness of PANC1 (Figure 4A) and BxPC3 (Supplementary Figure S3) cells (p<0.05), while antibody mediated neutralization of IL-6 inhibited this effect to baseline levels (p<0.05). These data support a role for PSC secreted IL-6 as a critical factor associated with enhanced cellular invasion of PDAC cells.

In order to assess whether PSC-CM stimulates anchorage-independent PDAC growth, soft agar colony formation assays were conducted using PANC1 cells treated with serum free control medium or hPSC-CM (100μg protein/ml) with or without the addition of IL-6 neutralizing antibody (Figure 4B). Exposure to hPSC-CM significantly increased the size of colonies formed while blockade of IL-6 with the neutralizing antibody significantly reduced hPSC-CM induced colony size to baseline levels (p<0.05), suggesting that PSC secreted IL-6 plays a role in supporting tumor growth.

### PSC secreted IL-6 stimulates cell invasion of precursor mouse PanIN cells in a STAT3 dependent manner

**Figure 5:**
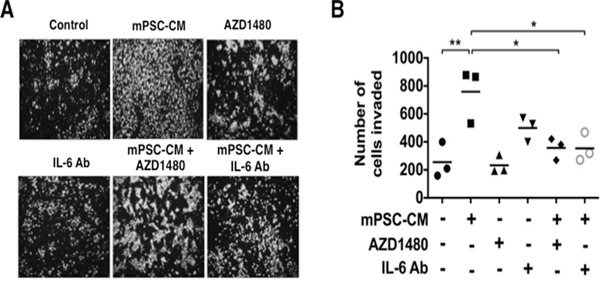
Mouse PSC-CM mediates activation of PanIN cell invasion through IL6-JAK-STAT3 signaling Mouse PanIN cells were treated with mPSC-CM (100μg total protein/ml) with or without AZD1480 (100 nmol/L) or IL-6 neutralizing Ab and analyzed for cell invasion. **A.** Images demonstrate representative pictures from each treatment group. **B.** PanIN cell invasion analysis results showed that either IL-6 neutralizing Ab or AZD1480 significantly blocked mPSC-CM induced activation of cell invasion. * – *P* < 0.05; ** – *P* < 0.01.

PanINs are precursor lesions of PDAC. Having shown that PSC secreted IL-6 enhances the tumorigenicity of PDAC cells via STAT3 signaling, we next sought to determine if IL-6 exposure can stimulate the invasive capabilities of non-invasive PanIN cells. Exposure of PanIN cells to mPSC-CM significantly enhanced cell invasion (Figure 5A). Interestingly, the enhanced and sustained phosphorylation of STAT3 seen with exposure to mPSC-CM (Figure 1B) correlated with the increased cell invasiveness induced by exposure to mPSC-CM. The invasive effect was significantly inhibited with an IL-6 neutralizing antibody (Figure 5B). To further determine if mPSC-CM mediated cell invasion was dependent on STAT3 activity, PanIN cells were treated with the JAK/STAT3 inhibitor AZD1480. Treatment with AZD1480 resulted in significantly attenuated mPSC-CM induced invasion of PanIN cells to control levels (Figure 5B). These results confirm that PSC secreted IL-6, enhances the invasive capacity of pre-cancerous PanIN cells in a STAT3 dependent manner. Our results further emphasize the tumor-stromal cross-talk that exists between PSCs and tumor cells, which not only enhances tumor progression of PDAC cells, but also stimulates the invasive capabilities of non-malignant PanIN lesions.

## DISCUSSION

We have previously shown that activated STAT3 is a biomarker and a key mediator of therapeutic resistance in PDAC [1, 2] [26]. In this study, we demonstrate a positive correlation expression of phosphorylated STAT3 in the progression of pancreatic neoplasia from normal ductal cells to precancerous PanIN cells to PDAC (Figure 1A); supporting the significant role that STAT3 plays in the progression of PDAC. Importantly, tumor associated PSCs have clearly been demonstrated to play a key role in PDAC-associated fibrogenesis by secreting components of the extracellular matrix (ECM) and tumor associated stroma [27, 28]. We have previously shown that STAT3 inhibition results in remodeling of the tumor stroma that is associated with increased drug delivery and cytotoxic therapeutic response of PDAC tumors in an aggressive mouse model of PDAC [20], further validating STAT3 as a novel and viable therapeutic PDAC target [3, 6, 9, 29–32].

Our current study shows that serum from KPC and PKT mice have higher levels of serum IL-6 then their control KC and PK counterparts respectively. Furthermore, IL-6 secreted from PSCs stimulates STAT3 signaling in both the mouse pancreatic precursor, noninvasive PanIN cells as well as in primary human PDAC cell lines. Secretion of IL-6 by PSCs directly enhances the tumorigenic potential of PanIN cells and stimulates progression of pre-neoplastic lesions to an invasive phenotype in a STAT3 dependent manner. This important finding defines a novel mechanism of PSC secreted IL-6 mediated activation of STAT3 signaling, as a critical regulator of tumor initiation and progression of PDAC.

This study further highlights the importance of cross-talk signaling processes and functional cell-ECM communication between the neoplastic cells and their microenvironment. This tug-of-war allows the dense stroma surrounding a growing tumor to recruit helper PSCs as well as other protective cell types of cellular immunity providing an environmentally supported transition into a more aggressive and metastatic phenotype. As we demonstrate, IL-6 plays a fundamental role in this process (Figures 2, 3, 4 and 5). IL-6 secreted from either human PSCs or mouse PSCs (Figure 2), appears to be a key factor that mediates the cross-talk between PSCs and PDAC cells. Neutralization of IL-6 with a ligand-specific antibody effectively reduces the overall free/active IL-6 levels in PSC-CM, blocks PSC-CM protein induced activation of STAT3 (Figure 3B), and abrogates PSC-CM enhanced growth and invasion of PDAC cells (Figures 4, 5). These findings reinforce the direct role of IL-6/STAT3 mediated PSC-PDAC interaction in promoting invasion and progression of pancreatic cancer. Additional IL-6 targeted therapeutics are under-going clinical trials for the treatment of renal cell cancer, prostate cancer, lymphoma and multiple myeloma [33]. Our data provides important pre-clinical information and rationale for targeting IL-6/STAT3 signaling in pancreatic cancer.

Accumulating evidence supports the notion that the tumor-associated stroma not only causes inefficient drug delivery, but also creates a cytokine and growth factor enriched microenvironment, which often results in enhanced proliferation and invasion of PDAC [6, 9, 20]. PSCs have been shown to induce PDAC cell resistance to chemotherapy [34, 35], which may be partly mediated by PSC secreted factors. Our data confirms the role of PSC secreted IL-6 in supporting the initiation and progression of PDAC. Therefore, therapeutic targeting of PSCs and/or IL-6 may provide a novel approach for anti-fibrotic and anti-neoplastic therapy, which could suppress IL-6 induced STAT3 activation and contribute to reduced invasiveness and tumorigenicity of PDAC. Supporting our findings, clinical studies have shown that elevated IL-6 serum levels correlate with worse patient outcomes [13, 36], and targeting IL-6 signaling with a neutralizing antibody has shown promising results in a clinical trial for the treatment of ovarian cancer [33].

Collectively, our results demonstrate for the first time that the IL-6/STAT3 signaling axis is a key element of tumor-stromal interaction between PSCs and tumor cells, playing a critical role in the progression from pre-neoplastic PanINs to PDAC. These findings demonstrate a novel role of PSCs in supporting an inflammatory TME and extend the evidence of crosstalk between the tumor stroma and diseased epithelium. Novel therapies focusing on both of these cell types and the TME should achieve better therapeutic responses.

## MATERIALS AND METHODS

### Cell lines, cell culture and reagents

Mouse PanIN cell line derived from the *LSL-Kras^G12D/+^; Pdx1^Cre/+^* (KC) GEMM. Primary PDAC (PDA) and liver metastasis (LMP) cell lines derived from the *LSL-Kras^G12D/+^; Trp53^R172H/+^; Pdx1^Cre/+^* (KPC) mouse models of PDAC [22] (kindly provided by Dr. Andrew Lowy, UC San Diego, CA) and maintained as previously described [37]. *Ptf1a^Cre/+^;LSL-Kras^G12D/+^* (PK) and *Ptf1a^Cre/+^;LSL-Kras^G12D/+^;Tgfbr2^flox/flox^* (PKT) mice were maintained as previously described [20]. The immortalized human pancreatic ductal cell line HPDE6-E6E7 (H6c7) was kindly provided by Dr. M.S. Tsao [38] and was maintained in in keratinocyte growth media (Invitrogen, Carlsbad, CA) supplemented with human epidermal growth factor and bovine pituitary extract. Human PDAC cell lines MiaPaCa2, PANC1 and BxPC3 were obtained from the American Type Culture Collection (ATCC). Tumor cells were maintained according to ATCC guidelines. Immortalized mouse PSCs and human PSCs were a gift from Dr. Anna Means (Vanderbilt University, Nashville, TN) and Dr. Ralf Jesnowski (German Cancer Research Center, Germany), respectively [18, 19]. AZD1480 was provided by AstraZeneca (Wilmington, DE). For *in vitro* experiments, AZD1480 was dissolved in 100% DMSO to prepare a 10 mM stock and stored at −20°C. Total STAT3 (124H6) and phospho-STAT3 (Y705) antibodies were purchased from Cell Signaling Technology (Denver, MA).

### Enzyme-linked immunosorbent assay (ELISA)

Serum levels of IL-6 was measured using human and mouse IL-6 Quantikine ELISA Kit (R&D Systems Inc, Minneapolis, MN) according to the manufacturer's instructions.

### Collection of human and mouse PSCs-conditioned medium (CM)

Human PSCs (hPSCs) and mouse PSCs (mPSCs) were grown on culture flasks until 70% to 80% confluent and then cultured in serum-free medium (SFM) for 24 hours at 37°C. Conditioned medium (CM) was collected separately from hPSCs and mPSCs and stored at −80°C in multiple rounds. Human and mouse collections were separately pooled. PSCs were maintained in culture with fresh media added twice weekly. CM and culture medium was concentrated using Millipore centriprep centrifugal filters (EMD Millipore, Billerica, MA) with 10k cut off and centrifuged at 2000 rpm, 4°C to concentrate. The concentrated CM and culture medium was quantified for the protein concentration and the aliquots were stored at −80°C. In the following experiments, PSC-CM and culture medium protein was diluted with serum-free medium (SFM) as indicated to evaluate the effect of PSCs secretions on the mouse PanIN and human PDAC cells.

### Western blot analysis

Western blot analysis was performed using standard methods previously described [39]. In brief, after treatment, cells were washed and scraped off the culture dishes. Cell pellets were collected by centrifuge and lysed in RIPA buffer (0.1% SDS, 50 mMTris HCl, 150 mMNaCl, 1% NP-40, and 0.5% Na deoxycholate) with protease inhibitor cocktail (Sigma, St. Louis, MO) and PhosSTOP phosphatase inhibitor (Roche, Indianapolis, IN, USA). Cell lysates were sonicated and centrifuged to collect supernatant. The collected supernatant was quantified for protein concentration measurements by Bio-Rad protein assay kit (Bio-Rad, Herculus, CA). The same quantities of protein were loaded from all the samples onto a SDS-PAGE gel. After running the gel, the proteins were transferred to a PVDF membrane and probed for the proteins of interest. Immunoblots were quantified using ImageJ and analysis was performed with Prism software (Graphpad Software Inc., La Jolla, CA).

### IL-6 neutralization assay

The IL-6 level in PSC-CM protein was determined by using a commercial ELISA according to the manufacturer's instructions (R&D Systems, Minneapolis, MN). The neutralization was performed at 1:400 ratios of IL-6 antibody (Abcam, Cambridge, MA) and PSC-CM protein (50 μg/ml) with the mixture incubation at 4°C overnight on a rocker.

### Cell invasion assay

Cell inserts (8.0 μm pore size membrane; Corning) were first coated with Matrigel (BD Biosciences, San Jose, CA) at a dilution of 3.3 ng/ml in SFM. Subsequently, 3 × 10^4^ cells, previously deprived of serum (0.1%) for 24 hours, were seeded on the Matrigel in the upper chamber in SFM, while the lower chamber contained media with PSC-CM protein with/without IL-6 neutralizing antibody and/or AZD1480. Standard culture medium was collected as detailed above and was used as control medium. After 48 hours, non-motile cells at the top of the filter were removed and the cells in the bottom chamber were fixed with methanol and stained with DAPI and counted using immunofluorescence microscopy. Results represent the average number of cells in 2 or 3 fields per membrane in triplicate inserts.

### Soft agar assays

5×10^4^ cells were suspended in media containing 0.33% Select Agar (Invitrogen, Carlsbad, CA) and plated on a bottom layer of media containing 0.5% Select Agar. Plates were incubated at 37°C for 2-3 weeks prior to imaging. IL-6 neutralizing antibody and/or hPSC-CM, and/or mPSC-CM protein was added directly into the top agar layer during plating. Colonies were photographed and quantified using ImageJ and analysis was performed with Prism software.

### Statistical and correlation analysis

Descriptive statistics including mean values and s.d. were calculated. Each treatment group was compared using one-way ANOVA and Tukey's multiple comparison test using Prism software (Graphpad Software Inc., La Jolla, CA). Results are shown as values of mean ± s.d. unless otherwise indicated. Pearson's and Spearman's correlation analysis were conducted in Stata 14 (StataCorp LP, College Station, TX).

## SUPPLEMENTARY MATERIALS FIGURES



## Abbreviations

PDAC: Pancreatic ductal adenocarcinoma
PSCs: pancreatic stellate cells
hPSCs: human pancreatic stellate cells
mPSCs: mouse pancreatic stellate cells
STAT3: signal transducer and activator of transcription 3
MDSCs: myeloid derived suppressor cells
TME: tumor microenvironment
PanIN: pancreatic intraepithelial neoplasia cell line
LMP: liver metastases line
H6c7: human pancreatic ductal epithelial cell line
