# Supplementary material for: Pancreatic stellate cell secreted IL-6 stimulates STAT3 dependent invasiveness of pancreatic intraepithelial neoplasia and cancer cells
Source: Oncotarget. 2016 Sep 1;7(40):65982–92. doi: 10.18632/oncotarget.11786 (PMC5323208; doi:10.18632/oncotarget.11786)
Supplement: Supplementary file 1 [file oncotarget-07-65982-s001.pdf]

Pancreatic stellate cell secreted IL-6 stimulates STAT3 dependent invasiveness of pancreatic intraepithelial neoplasia and cancer cells

SUPPLEMENTARY FIGURES

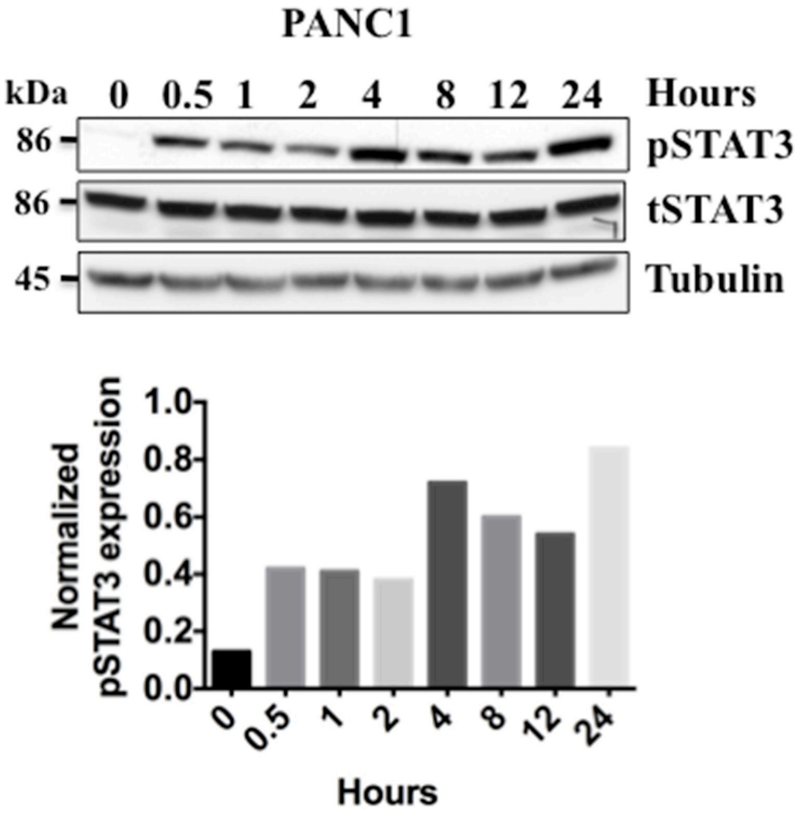

Supplementary Figure S1: Human PSC-CM (hPSC-CM) treatment activates STAT3 in PDAC cells. Exposure to concentrated hPSC-CM (100µg total protein/ml) results in activation of STAT3 (pSTAT3) in human PANC1 cells in a time-dependent manner. Tubulin was probed as a loading control. Densitometry analyses of immunoblots for pSTAT3 level normalized to tSTAT3 are shown in the graph.

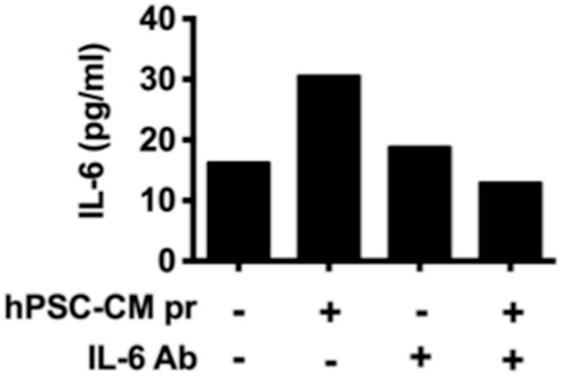

Supplementary Figure S2: Analysis of IL-6 concentration after neutralization with IL-6 antibody (IL-6 Ab). The level of IL-6 in hPSC-CM protein was measured by ELISA to confirm the efficacy of IL-6 neutralization. hPSC-CM pr = hPSC-CM; Ab = Antibody.

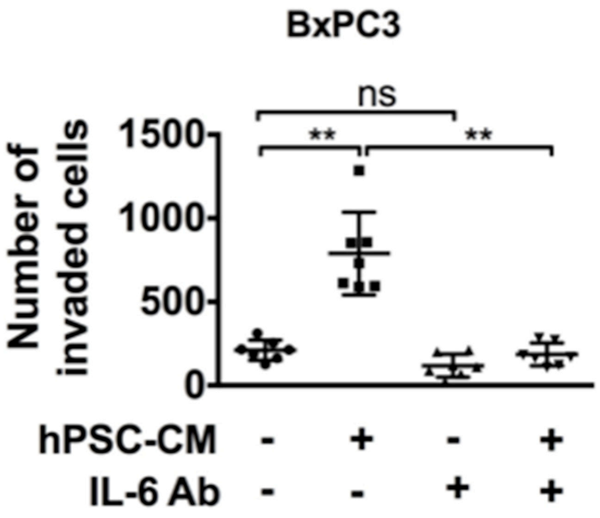

Supplementary Figure S3: Blocking IL-6 attenuates hPSC-CM induced cell invasion. BxPC3 cells were treated with hPSC-CM (100µg total protein/ml) with or without IL-6 neutralizing antibody (IL-6 Ab). Cell invasion was analyzed as detailed in Materials and Methods. Cell invasion results represent the average number of cells in seven fields for each of three separate inserts. ** – $P < 0.01$; ns – non-significant.
